# Prompt Outpatient Care For Older Adults Discharged From The Emergency Department Reduces Recidivism

**DOI:** 10.5811/westjem.2020.8.47276

**Published:** 2020-10-20

**Authors:** Phillip D. Magidson, Jin Huang, Emily B. Levitan, Andrew O. Westfall, Orla C. Sheehan, David L. Roth

**Affiliations:** * Johns Hopkins University School of Medicine, Department of Emergency Medicine, Baltimore, Maryland; † Johns Hopkins University School of Medicine, Division of Geriatric Medicine and Gerontology, Baltimore, Maryland; ‡ University of Alabama at Birmingham School of Public Health, Department of Epidemiology, Birmingham, Alabama; § University of Alabama at Birmingham School of Public Health, Department of Biostatistics, Birmingham, Alabama

## Abstract

**Introduction:**

Older adults present unique challenges to both emergency clinicians and health systems. These challenges are especially evident with respect to discharge after an emergency department (ED) visit as older adults are at risk for short-term, negative outcomes including repeat ED visits. The aim of this study was to evaluate characteristics and risk factors associated with repeat ED utilization by older adults.

**Methods:**

ED visits among participants in the Reasons for Geographic and Racial Differences in Stroke (REGARDS) study between 2003–2016 were examined using linked Medicare claims data to identify such visits and resulting disposition. Multilevel proportional hazards models examined associations of age, comorbidity status, race, gender, Medicaid dual eligibility status, social support characteristics (living alone or caregiver support), and use of ambulatory primary and subspecialty care with repeat ED utilization.

**Results:**

Older adults discharged from the ED seen by a primary care provider (hazard ratio [HR] = 0.93, confidence interval [CI], 0.87–0.98, p = 0.01) or subspecialist (HR = 0.91, CI 0.86–0.97, P <0.01) after the ED visit were less likely to return to the ED within 30 days compared to those who did not have such post-ED ambulatory visits. Additionally, comorbidity (HR =1.14, 95% CI, 1.13–1.16, P <0.01) and dual eligibility for Medicare and Medicaid (HR = 1.34, 95% CI, 1.20–1.50, p<0.01) were associated with return to the ED within 30 days. Those who were older (HR = 1.10, 95% CI, 1.05–1.15), had more comorbidities (HR = 1.17, 95% CI 1.15–1.18), Black (HR = 1.23, 95% CI, 1.14–1.33,P <0.01), and dually eligible (HR =1.23, 95% CI, 1.14–1.33, P <0.01) were more likely to return within 31–90 days after their initial presentation. The association of outpatient visits with repeat ED visits was no longer seen beyond 30 days. Patients without a caregiver or who lived alone were no more likely to return to the ED in the time periods evaluated in our study.

**Conclusion:**

Both primary care and subspecialty care visits among older adults who are seen in the ED and discharged are associated with less frequent repeat ED visits within 30 days.

## INTRODUCTION

The unique characteristics and needs of older adults present numerous challenges to the healthcare system that serves them, particularly in the fast-paced, high-resource setting of the emergency department (ED). Compared to younger patients, geriatric patients use the ED at disproportionally higher rates.^1^–^3^ Older patients seen in the ED are more likely to have extended lengths of stay, higher resource utilizations during their stays, and are more than three times as likely to be admitted to the hospital and five times more likely to be admitted to the intensive care unit, compared to younger patients.^2^–^6^ The increased cost of acute care services is one of the highest drivers of Medicare spending. Shifting this expensive, inpatient care to the post-acute and outpatient setting is one way to reduce healthcare spending; however, discharging older patients after an ED visit is not without risk.

Older patients who are treated in the ED and discharged back to the community have considerably more repeat ED visits that are associated with increased morbidity, mortality, and healthcare costs.^7^–^9^ Factors associated with repeat ED visits have not been thoroughly identified. Despite many emergency clinicians working to establish outpatient appointments prior to discharge, some smaller, single-center studies suggest outpatient follow-up after ED discharge may not reduce future ED utilization and repeat visits.^10^,^11^

With more than 20 million ED visits by patients over the age of 65 and the continued growth in this segment of the population, it is imperative that the healthcare system implement policies and practice guidelines that establish high-quality, low-cost care for geriatric patients seen in the ED. A shift to ambulatory care settings from the ED and a reduction in ED recidivism is likely to be one mechanism by which to achieve such a goal. As an initial step in helping to identify mechanisms for the delivery of higher value care, emergency clinicians, health system administrators, and policy makers would benefit from further identifying geriatric patients at particularly high risk for unplanned, return ED visits and factors associated with such events.

## METHODS

We extracted data from participants enrolled in the national REasons for Geographic and Racial Differences in Stroke (REGARDS) study database. REGARDS is a national cohort study that was designed to identify causes of both regional and racial disparities in stroke incidence. Due to its rich data collection methods, large sample size and linkage to Medicare claims, the REGARDS study has been used to examine numerous medical conditions and procedures beyond stroke. Additional details about the enrollment and data collection procedures in the REGARDS study have been described elsewhere.^12^

Potential participants for the REGARDS study were randomly sampled from a commercially available nationwide list of names with a corresponding telephone number and address. This list was purchased from a telecommunications company (Genesys Inc.. Daly City, CA). Eventual participants were 45 or older at the time of enrollment and of either Black or White race, with oversampling of the “stroke belt” region (Southeastern United States). Those determined to be eligible for enrollment had a baseline telephone interview and in-home visit. Every six months, follow-up telephone interviews were conducted with inquiries about outpatient- and hospital-based medical services. All participants in REGARDS provided written informed consent for researchers to obtain their health records, including electronic records such as Medicare claims files.^13^,^14^

**Population Health Research Capsule**

What do we already know about this issue?

*Repeat ED visits among older adults are associated with increased morbidity and healthcare costs*.

What was the research question?

Do primary care or subspecialty outpatient visits after an index ED visit, reduce recidivism among older adults?

What was the major finding of the study?

*Prompt outpatient care after an initial ED visit is associated with lower rates of repeat ED visits within 30 days, but this effect is lost beyond 30 days*.

How does this improve population health?

*For older adults discharged from the ED, the arrangement of prompt outpatient care prior to discharge may lead to higher value care among this patient demographic*.

The REGARDS database offers researchers numerous social (eg, caregiver support, marital status, household income, and education) and medical (eg, chronic medical conditions, surgical history, medication usage, and alcohol/tobacco usage) characteristics of enrolled patients as well as linked Medicare claims for the large proportion of participants enrolled in fee-for-service (FFS) Medicare. Moreover, this database is representative of the US population older than 65 with FFS coverage.^15^ All procedures were approved by the institutional review boards of participating institutions.

Medicare claims for ED visits were examined from 2003 to 2016. We identified patients with continuous FFS Medicare coverage in the preceding year and at least one ED visit resulting in discharge. Subsequent ED visits made within 1–30 and 31–90 days after the initial ED visit discharge were also identified for patients who had survived to 30 and 90 days, respectively, and had FFS Medicare coverage during those time periods. The unit of analysis was the ED visit nested within individual participants. Many participants contributed multiple ED visits to the analysis.

Demographic data including age, race, gender, caregiving availability, marital status, other social support and self-reported health data including disease history and health-related quality of life were obtained from REGARDS from a computer-assisted telephone interview (CATI) conducted at entry into the REGARDS study. Other predictors such as Charlson Comorbidity Index (CCI) and Medicaid dually eligible status were obtained for all patients included from the linked Medicare claims data using procedures previously implemented by our team.^13^ We identified outpatient visits by Current Procedural Technology codes specific to outpatient or home service provider care. Primary and subspeciality care was classified based on Centers for Medicare & Medicaid Services’ provider speciality codes.

We used descriptive analyses to quantify the prevalence of ED visits and repeat ED visits within the 1–30 day and 31–90 day follow-up periods. We used multilevel Cox proportional hazards analysis, with the ED visit resulting in discharge as the primary unit of analysis, nested within individual participants.^16^ A robust sandwich estimate of the covariance matrix was used to account for the clustering of qualifying ED visits within participants.^17^ Race, gender, marital status, caregiver availability and living alone – assessed at entry into the REGARDS study – were treated as time-invariant, person-level covariates. We treated age, CCI, and dual eligibility as covariates that are fixed for each visit but may vary across visits. Outpatient care visits were treated as time-varying covariates within each follow-up period.^18^

## RESULTS

**Figure 1:**
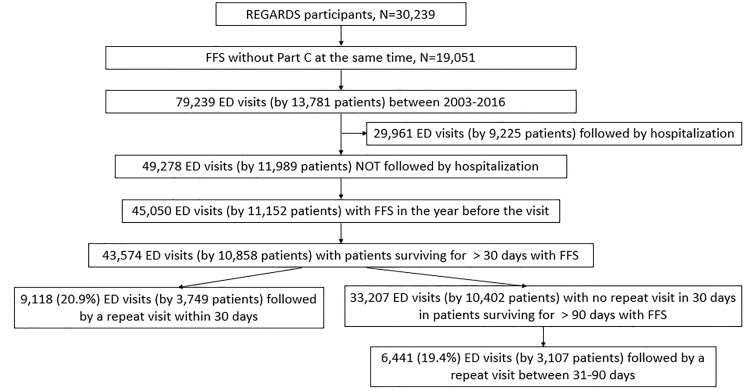
REGARDS participants between 2003–2016 included in analysis. *REGARDS*, REasons for Geographic and Racial Differences in Stroke; *ED*, emergency department, *FFS*, Medicare fee for service.

Figure 1 provides a schematic representation of those patients included in our analysis. A total of 30,239 individual participants were enrolled into the REGARDS study with 19,051 ever having FFS (Medicare Parts A and B) but no health maintenance organization (Medicare Part C) coverage at the same time. For these 19,051 patients, 79,239 ED visits were observed in the Medicare claims for 13,781 patients who had at least one such visit. Of those visits, 49,278 visits (by 11,989 patients) did not result in hospitalization. Of those that did not result in hospitalization, 96% resulted in a discharge home. Those patients who had continuous Medicare coverage in the preceding year accounted for 45,050 total visits by 11,152 patients. Of these patients, 10,858 (who accounted for 43,574 visits) survived at least 30 days and continued to have Medicare FFS coverage during that time. Among these patients, the mean number of ED visits per patient was 4.01 (standard deviation = 5.0) with a median of 2.0 (Q1=1.0, Q3=5.0). Further participant characteristics at the time of the first ED visit are included in Table 1.

In the 30-day follow-up group, 20.9% (n = 9,118) of ED visits were followed by a repeat visit. An additional 19.4% (n = 6,441) of the initial ED visits were followed by a repeat ED visit within 31–90 days. For the entire 90-day period, which includes only those patients who survived from day 1 through day 90, there were 14,898 repeat ED visits of 41,664 initial visits with a return rate of 35.8%. Of older adults seen in the ED for an initial visit and then discharged, those patients with a higher CCI (hazard ratio [HR] =1.14, 95% confidence interval [CI], 1.13–1.16, *P* <0.01) and who were dually eligible for Medicare and Medicaid (HR = 1.34, 95% CI, 1.20–1.50, *P* <0.01) were more likely to have returned to the ED within 30 days. With respect to age, gender, race (Black vs White) or marital status, there were no significant differences in return ED visits at 30 days.

Older patients (HR = 1.10, 95% CI, 1.05–1.15), those with more comorbidities (HR = 1.17, 95% CI. 1.15–1.18) as well as dually eligible Medicare and Medicaid beneficiaries (1.49, 95% CI, 1.35–1.64) continued to be more likely to return to the ED within 31–90 days (all *P* values <0.01). During this follow-up period, however, Black patients were found to be more likely than Whites to return to the ED (HR =1.23, 95% CI, 1.14–1.33, *P* <0.01). Gender as well as marital status, as in the 30-day follow-up group, were not associated with an increase in return ED visits.

From an outpatient medical resource standpoint, both primary care (HR = 0.93, CI, 0.87–0.98, *P* = 0.01) and subspecialty care (HR = 0.91, CI, 0.86–0.97, p<0.01) was associated with reduced 30-day repeat ED visits. However, within the 31–90 days follow-up period, this association was no longer seen for either primary care or subspecialty care. For patients who did not return to the ED within 30 days, the average time from ED discharge to primary care visit and subspecialty visit was 10.2 and 11.1 days, respectively. With respect to social support resources, those patients without an available caregiver or who reported living alone, were no more likely to return to the ED than those with such resources for both the 30-day and 31–90 day time periods respectively (Table 2).

## DISCUSSION

Within a population of older adults seen in the ED, we found that 20.9% of initial ED visits resulting in discharge were followed by another ED visit within 30 days. For all initial ED visit by patients who survived to 90 days, 35.8% were followed by another ED visit within 90 days. However, older adults who saw a primary care provider (PCP) or subspecialist after the index ED visit were significantly less likely to have a repeat ED visit within 30 days compared to those patients who did not have an ambulatory outpatient visit. These findings were not seen among older adults beyond 30 days, suggesting that prompt outpatient follow-up — that is, follow-up within 10–12 days — is more beneficial than delayed outpatient follow-up. These findings are consistent with similar studies looking at the utility of prompt vs delayed primary care follow-up, albeit in a younger patient population and within the confines of a specific “rapid-access-to-primary-care” program.^19^ Other specific characteristics that impact the likelihood of ED recidivism among older Medicare beneficiaries include advanced age and dual eligibility status, as well as comorbidity status as measured by the CCI.

Some studies have examined the association between social factors and ED recidivism. Specifically, veterans with chronic obstructive pulmonary disease were found to be more likely to return to the ED within two weeks if they were widowed, separated, or divorced.^20^ A related study showed older men living alone were more likely to return to the ED within 90 days compared to older men living with someone else.^21^ Other social factors such as the role caregivers play in ED usage is well documented in the pediatric literature but less so in older patients. One study that examined ED use after stroke demonstrated an association between caregiver support and a reduction in ED visits.^13^ Overall reported poorer health status, lower education level, and lower household income have been associated with an increase in ED use among all patients. However, these associations, specifically among older patients and return ED visits, have not been sufficiently demonstrated.^22^

In our study, social factors such as the lack of an identified caregiver and living alone were not associated with an increase in ED visits at 30 days or between 31–90 days. This may relate to the characteristics of older adults who are receiving help from a caregiver. Specifically, these individuals may have more complicated medical conditions or be more likely to require assistances with self-care activities compared to older adults without such caregiver support.^23^,^24^ This may predict a population that is more likely to require hospital-based services such as emergency care and thus offset any benefit having a caregiver may offer.

There is considerable controversy in the literature with respect to the use of the ED by Black patients compared to White patients with some studies suggesting an increased use among Blacks while other studies showing similar use patterns across races.^25^–^28^ As in the previously published literature, our study showed mixed results with no difference in repeat visits within 30 days. However, between the 31- and 90-day follow-up time period, Blacks were more likely to return to the ED compared to White patients.

Although reassuring timely outpatient primary and subspecialty care offers protective benefits for Medicare beneficiaries discharged from the ED, improvements in transitional care between the ED and ambulatory providers must also be made. Currently there are no standardized communication handoff tools used by emergency providers to ensure consistent communication with their primary care or other ambulatory colleagues.^29^ This lack of standardized communication gap is appreciated by both emergency clinicians and PCPs alike and is associated with increased ED length of stay as well as consuming time and resources in the primary care setting.^30^,^31^

To our knowledge, the protective nature of both primary care and subspecialty follow-up visits after an ED discharge in older adults has not been described before with respect to ED recidivism. This has very important pragmatic implications for practicing emergency clinicians. Moreover, the findings are of interest to healthcare administrators and payers in an environment where there is continued pressure to provide lower cost outpatient services in lieu of expensive, hospital-based care.

## LIMITATIONS

Limitations of our study include reliance on both claims data as well as on self-reported survey results. With respect to claims data, our analysis looked at the FFS Medicare population and may not be generalizable to all older adults. Additionally, healthcare claims are generated for payment purposes and may not totally capture the specific care a patient received. It is possible, for example, that some patients received outpatient clinical services after an ED visit, which were not reflected in the claims for payment. However, given that a provider’s reimbursement for services would be adversely impacted by not filing a claim, we feel the number of outpatient visits that did not generate a claim would be very small.

Additionally, our study primarily looked at community-dwelling older adults who were discharged from the ED, which would exclude those who transitioned to skilled nursing facilities or other short-term rehabilitation units; however, we would anticipate this number to be low and therefore unlikely to change our results. It should further be noted that the residential status and the availability of a caregiver was obtained at the time of REGARDS enrollments, not necessarily at the time of ED visit, and such status could have changed over time.

## CONCLUSION

Prompt primary care and subspecialty care for older adults who were seen in the ED and discharged home was associated with lower rates of subsequent, repeat ED visits within 30 days. This protective effect is lost beyond 30 days, suggesting outpatient follow-up should occur within 10–12 days to prevent ED recidivism.

## Figures and Tables

**Table 1:** Participant characteristics at first ED emergency department visit from REGARDS.*

Variable	N = 10,858
Age at 1st ED visit, mean (SD)	73.36 (7.91)
CCI, mean (SD)	1.43 (1.80)
Female, n (%)	5,857 (53.94)
Black, n (%)	3,993 (36.77)
Dual eligible, n (%)	1,438 (13.24)
Marital status, n (%)	
Married	6,215 (57.24)
Divorced	1,418 (13.06)
Single	423 (3.90)
Widowed	2,558 (23.56)
Other	244 (2.25)
Available caregiver, n (%)	8,714 (80.25)
Living alone, n (%)	3,249 (29.93)

* *REGARDS*, REasons for Geographic and Racial Differences in Stroke; *ED*, emergency department; *CCI*, Charlson Comorbidity Index; *SD*, standard deviation.

**Table 2:** Multivariable Cox proportional hazard models on time to repeated emergency department visit.

Variable	30 days (n = 10,858, 43,574 ED visits)		30–90 days (n = 10,402, 33,207 ED visits)	

	Hazard ratio (95% CI)	P-value	Hazard ratio (95% CI)	P-value
Age at index ED visit (10-year)	1.04 (0.99, 1.09)	0.16	1.10 (1.05, 1.15)	<0.01
CCI (1 unit)	1.14 (1.13, 1.16)	<0.01	1.17 (1.15, 1.18)	<0.01
Female vs male	0.96 (0.89, 1.04)	0.36	0.98 (0.90, 1.07)	0.68
Black vs White	1.02 (0.94, 1.11)	0.68	1.23 (1.14, 1.33)	<0.01
Dual eligible	1.34 (1.20, 1.50)	<0.01	1.49 (1.35, 1.64)	<0.01
Marital status				
Divorced vs married	1.04 (0.89, 1.21)	0.66	1.06 (0.91, 1.22)	0.46
Other vs married	0.97 (0.78, 1.20)	0.77	1.16 (0.93, 1.45)	0.20
Single vs married	1.01 (0.83, 1.23)	0.89	1.07 (0.88, 1.30)	0.51
Widowed vs married	1.04 (0.91, 1.18)	0.56	1.05 (0.93, 1.18)	0.41
Available caregiver	0.94 (0.85, 1.04)	0.20	0.95 (0.87, 1.04)	0.26
Living alone	1.11 (0.99, 1.24)	0.09	1.06 (0.96, 1.17)	0.28
Primary care visit	0.93 (0.87, 0.98)	0.01	1.05 (0.99, 1.11)	0.13
Subspecialty visit	0.91 (0.86, 0.97)	<0.01	1.04 (0.99, 1.11)	0.14

*CI*, confidence interval; *ED*, emergency department; *CCI*, Charlson Comorbidity Index.
